# Hearing loss in outbred Hartley guinea pigs experimentally infected with Pichinde virus as a surrogate model of human mammarenaviral hemorrhagic fevers

**DOI:** 10.1080/21505594.2022.2087948

**Published:** 2022-06-26

**Authors:** Morgan Brisse, Claudia Fernández-Alarcón, Qinfeng Huang, Natalie Kirk, Mark R. Schleiss, Yuying Liang, Hinh Ly

**Affiliations:** a Biochemistry, Molecular Biology and Biophysics Graduate Program, University of Minnesota, Twin Cities, Minnesota, USA; b Comparative and Molecular Biosciences Graduate Program, University of Minnesota, Twin Cities, Minnesota, USA; c Department of Pediatrics, School of Medicine University of Minnesota, Twin Cities, Minnesota, USA; d Department of Veterinary and Biomedical Sciences College of Veterinary Medicine, University of Minnesota, Twin Cities, Minnesota, USA

**Keywords:** Virus-induced hearing loss, Sensorineural hearing loss (SNHL), Pichinde virus, Lassa virus, Mammarenavirus, Arenavirus, guinea pigs

## Abstract

Lassa fever (LF) is a neglected tropical disease that is caused by Lassa virus (LASV), a human hemorrhagic fever-causing mammarenavirus. A notable sequela of LF is sensorineural hearing loss (SNHL) that can develop in about 33% of the patients. Animal models of LF-associated SNHL have been limited in size and scope because LASV is a biosafety level 4 (BSL4) pathogen that requires its handling in a high biocontainment laboratory. In this report, we describe the development of an alternative arenavirus hearing loss model by infecting outbred Hartley guinea pigs with a virulent strain (rP18) of the Pichinde virus (PICV), which is a guinea pig-adapted mammarenavirus that has been used as a surrogate model of mammarenaviral hemorrhagic fevers in a conventional (BSL2) laboratory. By measuring auditory brainstem response (ABR) throughout the course of the virulent rP18 PICV infection, we noticed that some of the animals experienced an acute but transient level of hearing loss. Cochleae of hearing-impaired animals, but not of controls, had demonstrable viral RNA by quantitative RT-PCR, indicating the presence of virus in the affected inner ear with no overt histopathological changes. In contrast, neither the outbred Hartley guinea pigs infected with a known avirulent strain (rP2) of PICV nor those that were mock-infected showed any evidence of hearing loss or viral infection of the inner ear. This is the first report of an immunocompetent small animal model of mammarenavirus-induced hearing loss that can be used to evaluate potential therapeutics against virus-induced hearing impairment under a conventional laboratory setting.

## Introduction

Lassa virus (LASV) is an Old World mammarenavirus (also known as an arenavirus) that is endemic to west Africa and causes a severe form of hemorrhagic disease known as Lassa fever (LF). It is estimated that as many as 300,000 infections and 5,000 deaths occur annually as a result of LF [1]. LF incubation period ranges from 7 to 21 days before the onset of disease signs [2] which typically begin with nonspecific symptoms such as fever, lethargy, and headache [3,4]. A subset of patients will progress to severe LF with findings such as nausea, vomiting, renal and hepatic dysfunction, and hemorrhage, with the most severe cases eventually resulting in multi-system organ failure and death [4]. LF is included in the World Health Organization (WHO)’s list of diseases with the highest urgency for infectious disease research [5]. LF was designated as one of the WHO blueprint priority diseases because of its relatively high case load, the lack of available vaccines, and highly efficacious anti-viral treatments [6] and its pandemic potential. LASV has caused multiple disease outbreaks in several countries in western Africa and has been imported into other countries, such as the US, the EU, and Japan through means of international travel [7].

An under-addressed consequence of severe LF is the onset of sensorineural hearing loss (SNHL), which by some estimates may occur in up to 33% of LF patients [8]. SNHL differs from conductive hearing loss in that SNHL results from permanent damage to the auditory nerves and/or other essential anatomical structures necessary for sound transduction, whereas conductive hearing loss typically occurs because of a physical or inflammatory blockage of the ear canal and is usually transient in nature. SNHL has been well documented for several viral infections, including cytomegalovirus (CMV) [9–11] rubella [11] Zika, mumps [12], and Ebola virus [13], and has also been documented in certain other circumstances including drug overdoses [14–17] and autoimmune conditions [18] such as lupus [19]. SNHL is typically identified 10–15 days after the initial onset of LF disease signs or during the convalescence stage of the disease [20], with two documented cases having been identified during the acute stage [21]. The duration of SNHL is also unclear, although there have been documented cases of SNHL lasting up to 4 years [8,21]. The development of treatment and prevention options for LASV-induced SNHL is especially urgent given the decreased quality of life and social stigma that can result from permanent hearing loss caused by this virus infection [8,22,23].

Due to the inherent difficulties of studying the specific molecular mechanisms responsible for hearing loss in humans, animal modeling is necessary to conduct deeper and more regimented studies of LASV-induced hearing loss. Several animal models currently exist to model human LASV infection [24], but models that directly utilize LASV need to be handled in a high biocontainment laboratory because LASV is a BSL4-level pathogen. As such, previous hearing studies in LASV-infected animals have been limited by the relatively small sample sizes and the lack of a human-analogous hearing quantification methodology. Nevertheless, those studies found some evidence of hearing loss in some of the LASV-infected animals [25,26] as a result of inflammation and immune cell invasion in the cochlea [25] or in the surrounding support tissues [26] of nerve damage [25], and/or of the presence of LASV within the cochlea of some of the infected animals [25,27].

An alternative (surrogate) animal model of human mammarenaviral hemorrhagic fevers, including LF, that can be conducted in a conventional (BSL2) laboratory involves outbred Hartley guinea pigs infected with a virulent strain of the Pichinde virus (PICV) [28–30]. PICV is a New World mammarenavirus that was originally isolated from *Oryzomys albigularis* rodents in Colombia [29]. PICV was passaged successive times in guinea pigs and suckling mice to eventually generate a virulent form of the disease in guinea pigs and to produce the more pathogenic P18 strain of the virus [29]. Our laboratory has developed a reverse genetics system for the production of a recombinant P18 (rP18) virus that can produce a consistently and significantly higher level of morbidity and mortality in infected outbred Hartley guinea pigs as compared to those infected by the parental P18 virus [31]. Outbred Hartley guinea pigs systemically infected with rP18 virus develop many of the same disease signs as those of human LF patients, such as bodyweight loss, sustained fever, hepatic and blood dysfunction, and hemorrhage in some infected individuals [28–30], and can result in 90% lethality in young animals of less than 4 weeks of age [28]. As such, rP18 infection of outbred Hartley guinea pigs can be considered as an appealing surrogate animal model of human mammarenaviral hemorrhagic fevers, including LF. However, no hearing studies have previously been conducted using this convenient small animal model of human LF.

To this end, we set out to determine whether rP18-infected outbred Hartley guinea pigs can experience hearing loss in addition to other known disease signs that overlap with human mammarenaviral hemorrhagic fevers. We found that a significant portion of the outbred Hartley guinea pigs infected with rP18 experienced hearing loss, while outbred Hartley guinea pigs that have either been mock-infected or infected with a known avirulent rP2 virus did not experience hearing loss, as determined by auditory brainstem response (ABR). However, we did not find any consistent and significant histopathological changes in cochleae associated with hearing loss but were able to detect PICV RNAs in some hearing-impaired cochleae of the virulent rP18-infected animals.

## Materials and Methods

### Plasmids and Cells

The plasmids that contain the PICV genome and the methods used for generating rP18 and rP2 viruses have been described previously [31,32]. Baby hamster kidney cells (BHK21) and African green-monkey kidney cells (Vero) were cultured in minimal essential media (MEM) supplemented with 10% heat-inactivated fetal bovine serum (FBS) and 50 μg/mL penicillin-streptomycin. BSRT7–5 cells, which were obtained from K.-K. Conzelmann (Ludwig-Maximilians-Universität, Germany), were cultured in minimal essential media (MEM) supplemented with 10% heat-inactivated fetal bovine serum (FBS) and 50 μg/mL penicillin-streptomycin.

### Virus production and sequencing

The methods for generating rP18 and rP2 viruses have been described previously [31,32]. Supernatants containing these recombinant rPICVs were collected at 48 h and 72 h post plasmids transfection. The rPICVs were amplified once in BHK21 cells, and the titers of these virus stocks were determined by plaque assay and stored at −80°C until use. Viral RNAs were extracted from virus stocks (or infectious viral plaques) using the QIAamp Viral RNA kit (Qiagen, USA), amplified by RT-PCR and sequenced to confirm the identity of the virus stocks. The PICV sequencing primer had the sequence 5’-GGCATCAGCCAAGTCCTTTA-3’.

### Guinea pigs studies

We used the approved protocol of the Institutional Animal Care and Use Committee (IACUC) at the University of Minnesota to conduct animal studies. Briefly, outbred male Hartley guinea pigs were obtained from Elm Hill Breeding Labs (USA). Based on our extensive historical data and analysis [28,31,33,34], 1 × 10^6^ pfu (plaque-forming unit) of the virulent rP18 strain of PICV was used to inject intraperitoneally (IP) into 6–14-week old outbred Hartley guinea pigs (n = 8) to generate a uniform level of virulent PICV infection, whereas IP injections of 1 × 10^6^ pfu of the avirulent rP2 strain of PICV into each of the outbred Hartley guinea pigs (n = 6) were not known to cause any significant disease signs. Additionally, three outbred Hartley guinea pigs were injected with phosphate-buffered saline (PBS) as mock-infected controls. The animal’s bodyweights and rectal temperatures were monitored daily following the injections. The phenotypic differences between the rP2- and rP18-infected animals were described in our previous reports [28,31,33,34]. Blood was taken from the animals via toenail clip at four-day intervals following the virus infection and used for virus titration analysis by plaque assay. At 8 days post infection (8 dpi) for rP18 (n = 2), 12 dpi for rP18 (n = 2), 16 dpi for rP18 (n = 4) and PBS-mocked infection (n = 2) and 30 dpi for rP2 (n = 6) and for PBS-mocked infection (n = 1), animals were euthanized to collect organs and sera for viral titration analysis by plaque assay, viral RNA quantification by RT-qPCR, and histopathological analysis by hematoxylin and eosin (H&E) staining. rP18 animals were sacrificed at 4-day intervals starting at day 8 (which is when virus becomes consistently detectable in the serum of infected animals) until terminal point at day 16 to identify any potential cochlear pathology along the course of the infection. Two mock-infected animals were sacrificed at day 16 to serve as a control for the rP18 animals. The rP2-infected animals and the remaining mock-infected animals were euthanized on day 30 to detect any convalescence-onset hearing loss as seen with Lassa fever patients.

### Plaque assay

Vero cells were seeded into 6-well plates at 60%–70% confluency (4 × 10^5^ cells/well). In the following day, cells were infected with 500 µL of either serially diluted rPICV stocks or serum of virus-infected animals (in PBS) for 1 h at 37°C. After washing once with PBS, cells were incubated in MEM supplemented with 0.5% agar and cultured for an additional 4 days at 37°C and 5.0% CO_2_. Plaques were stained overnight with a diluted neutral red solution (0.01%) in 0.5% agar-MEM medium.

### Auditory brainstem response (ABR)

At 4-day intervals starting on the day of infection (day 0), hearing was measured in all animals by auditory brainstem response (ABR) method as previously described [9]. Briefly, the animals were anaesthetized with a combination of ketamine (30 mg/kg) and xylazine (3 mg/kg) delivered intramuscularly (IM). Following confirmation of anesthesia, subdermal needle electrodes (Life Sync Neuro, USA) were placed behind each auricle and at the vertex of the animal. The needle electrodes were fed by an Opti-Amp bioamplifier (Intelligent Hearing Systems, USA) with a 100,000 amplification gain and a 300-Hz to 5-kHz setting, with an average of 512 sweeps per bandpass filter. Rarefaction clicks (100 microseconds) were delivered into the ear by a plastic tube inserted into the external auditory canal at a rate of 29.3/s. Impedance was maintained at 3 kΩ or less. The average click responses were measured at 10 dB intervals between 0 and 80 dB. Thresholds were defined as the lowest intensity to yield a nonreproducible deflection in the evoked response traces for each frequency.

### Preparation of cochleae

Cochleae were prepared for tissue sectioning as described previously [35]. The *paries labyrinthicus* housing the cochlea was exposed after a dissection of the temporal bone bulla from the skull. Following the removal of the otic capsule, the left and right cochleae were fixed overnight in 4% paraformaldehyde (PFA) at 4°C followed by an overnight incubation in PBS at 4°C. Cochlea underwent decalcification for 2–3 weeks in the decalcification solution (10% EDTA in PBS) until the cochlea was translucent. Cochlea then underwent sucrose infiltration by washing in 10% sucrose for 30 minutes at room temperature followed by washing in 15% sucrose for 30 minutes at room temperature and an overnight incubation in 15% sucrose at 4°C. Cochlea was incubated in 1:1 15% sucrose/OCT compound (Andwin Scientific, USA) at 4°C for about 24 h, and then again overnight in degassed OCT at 4°C. Cochlea in OCT was snap frozen in liquid nitrogen the following day. Tissue sections were prepared by cutting 10 μm sections of the tissue in a cryostat. Hematoxylin and eosin (H&E) staining was performed by the veterinary diagnostic laboratory at the University of Minnesota.

### RNA extraction and RT-qPCR

Cochleae frozen in OCT were homogenized, and total RNA was extracted from the cochlea using TRIzol reagent (Life Technologies, USA). Single-stranded cDNAs of the bi-segmented PICV genome were generated using random hexamers (Invitrogen, USA) and the MMLV reverse transcriptase enzyme (Promega, USA) following the manufacturer’s protocol. Quantitative RT-qPCR was conducted using primers that were specific to the PICV nucleoprotein (NP) gene, whose sequence was conserved between the rP2 and rP18 strains, as well as primers that were specific for the cellular GAPDH as a control. The sequences of the primers used for RT-qPCR are as follows: PICV NP F 5’-CCCGGACAGAGAAATCCTTATG-3“+), PICV NP R 5’-CTCCCTTGAACTTGAGACCTTG-3”), Guinea pig GAPDH F 5’-ACAGTGACAGCCATTCTTCC-3“+), Guinea pig GAPDH R 5’-AGCCGAACTCATTGTCATACC-3”). The IQ SYBR Green supermix (Bio-Rad, USA) was used for the DNA amplification in a Bio-Rad CFX quantitative PCR machine (Bio-Rad, USA) at 95°C for 3 min, followed by 40 cycles of DNA amplification at 95°C for 10 s and 60°C for 30 s. The absolute concentration of viral RNA was determined by performing a standard curve using a PICV-NP containing plasmid [36] and was normalized to the tissue mass. Cochleae processed for RNA extraction are as follows: P18 animal 1 (left and right cochlea), animal 2 (left cochlea), animal 4 (left and right cochlea), animal 5 (left cochlea), animal 6 (left and right cochlea), animal 8 (left cochlea), P2 animal 2 (left and right cochlea), animal 3 (left and right cochlea), animal 4 (left and right cochlea), animal 5 (left and right cochlea), and animal 6 (right cochlea).

### Statistical analysis

For Figures 2(a–b), normalized weight and temperature were modeled using a linear mixed model with day (as a categorical variable), group, and the day/group interaction as fixed effects and animal as a random effect; overall pairwise tests between groups were computed using joint tests of the differences across all days. For Figure 2(a), day 0 was not included as all animals were normalized to have the same baseline value on day 0. For Figure 2(c), the statistical significance of the differences in the mean values was analyzed using an unpaired, two-tailed Student’s *t*-test for each individual time point. For Figure 3, the statistical significance was determined by Kruskal–Wallis one-way ANOVA by comparing the change in ABR threshold hearing levels from baseline at days 4–16 post infection between the rP18-, rP2-, and mock-infected animals. For Figure 4, correlation was determined by linear regression. For Figure 6, the statistical significance was determined by using Kruskal–Wallis one-way ANOVA comparing viral RNA copy numbers between cochleae from rP18-infected animals that had demonstrated hearing loss, cochleae from rP18-infected animals that had not demonstrated hearing loss, and rP2-infected animals. Student’s *t*-tests, ANOVA, and linear regression were performed using GraphPad PRISM (GraphPad Software, USA) and mixed model analysis was performed using R (R: A language and environment for statistical computing. R Foundation for Statistical Computing, Vienna, Austria. URL https://www.R-project.org/).

## Results

### Morbidity and viral kinetics of guinea pig infected with rP2 and rP18 viruses

**Figure 1.**
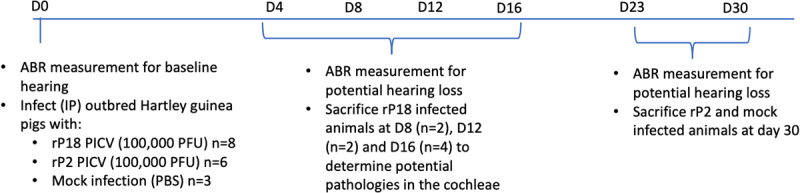
Experimental design. Figure shows timeline of experimental design for all experimental groups, including avirulent rP2 PICV infection, virulent rP18 PICV infection, and mock infection (PBS injection). Dates of hearing loss testing via the auditory brainstem response (ABR) method, of sample collection, and of animal sacrifice are indicated for different groups of animals.

**Figure 2.**
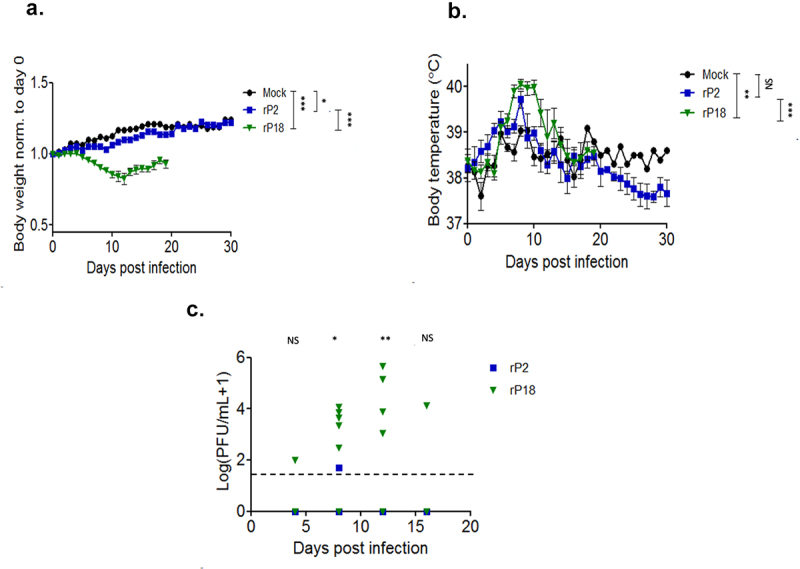
Morbidity, mortality, and viral kinetics of rP2- and rP18-infected outbred Hartley guinea pigs. Average bodyweights of virus-infected animals reported for each day following the infection and normalized to the bodyweight on the day of the infection (a). Average rectal temperatures in Celsius for each experimental group at each day of the infection (b). Serum’s viremia levels as determined by plaque assay (c). Statistical analyses were determined by mixed model analysis for Figure 2(a-b), and by Student’s *t*-test for Figure 2(c)2(c). For Figure 2(C), the statistical significance of the differences between rP18 and rP2 was analyzed using an unpaired, two-tailed Student’s *t*-test for each individual time point. *=p < 0.05, **=p < 0.01, ***=p < 0.001, “NS” non-statistically significance.

To investigate the initiation and development of hearing loss and other disease signs and mortality during the course of the virulent rP18 virus infection, some of the rP18-infected outbred Hartley guinea pigs were sacrificed at 8 dpi, 12 dpi, and 16 dpi, which coincided with the predetermined terminal point of the rP18 virus infection [28,31] as defined by bodyweight loss of 30% when compared to uninfected animals after taking normal weight gain into account, as well as high and sustained fever of >39.5°C As controls, outbred Hartley guinea pigs were injected with a known avirulent rP2 virus or with PBS (as mock infection). These animals were sacrificed at day 30 post-injection to investigate any possible convalescent-stage effects on hearing impairment (Figure 1). The rP18-infected animals experienced significantly more bodyweight loss (Figure 2(a)) and longer duration of fever (Figure 2(b)) than animals infected with the avirulent rP2 or with PBS injection (mock infection), which are consistent with our previous observations [28,37,38]. Serum’s viremia were also found to be at significantly higher levels in the rP18-infected animals than those in the rP2-infected animals at all timepoints examined (Figure 2(c)). Taken all together, these results indicate that we successfully reestablished a consistent surrogate model of LF by infecting outbred Hartley guinea pigs with the virulent rP18 virus that could be used for examining potential issue of hearing impairment.

**Figure 3.**
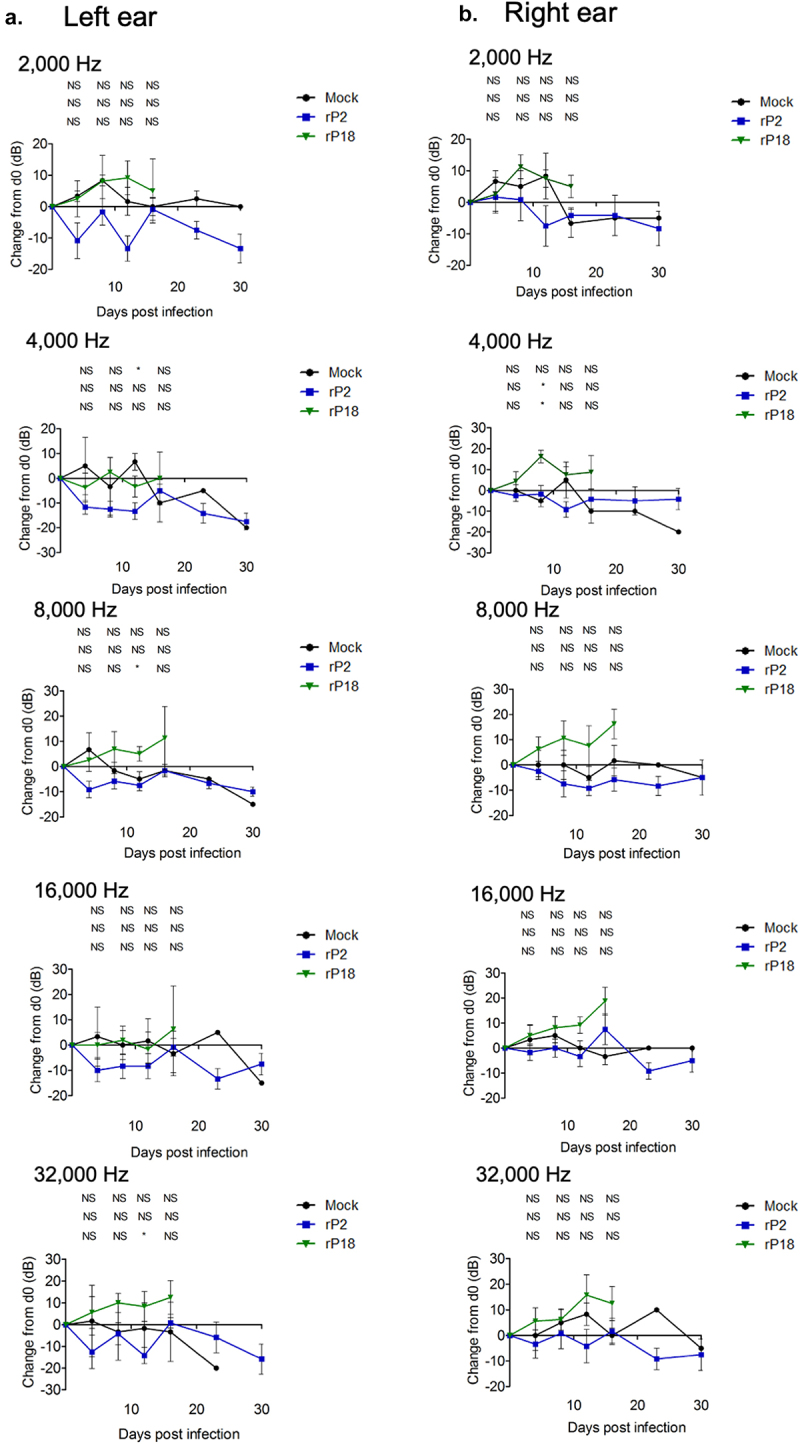
Virulent rP18-infected outbred Hartley guinea pigs experienced hearing loss. Figures are reported as the average changes in ABR thresholds from baseline decibel levels at day 0 for each experimental group in the left ear (a) and the right ear (b) when 2,000 Hz, 4,000 Hz, 8,000 Hz, 16,000 Hz, and 32,000 Hz of sound were used. Statistical analyses were performed using Kruskal–Wallis one-way ANOVA for each individual time point, comparing mock infection to rP2 infection (top), mock infection to rP18 infection (middle), and rP18 to rP2 infection (bottom). *=p < 0.05, **=p < 0.01, ***=p < 0.001, “NS” non-statistically significance.

**Figure 4.**
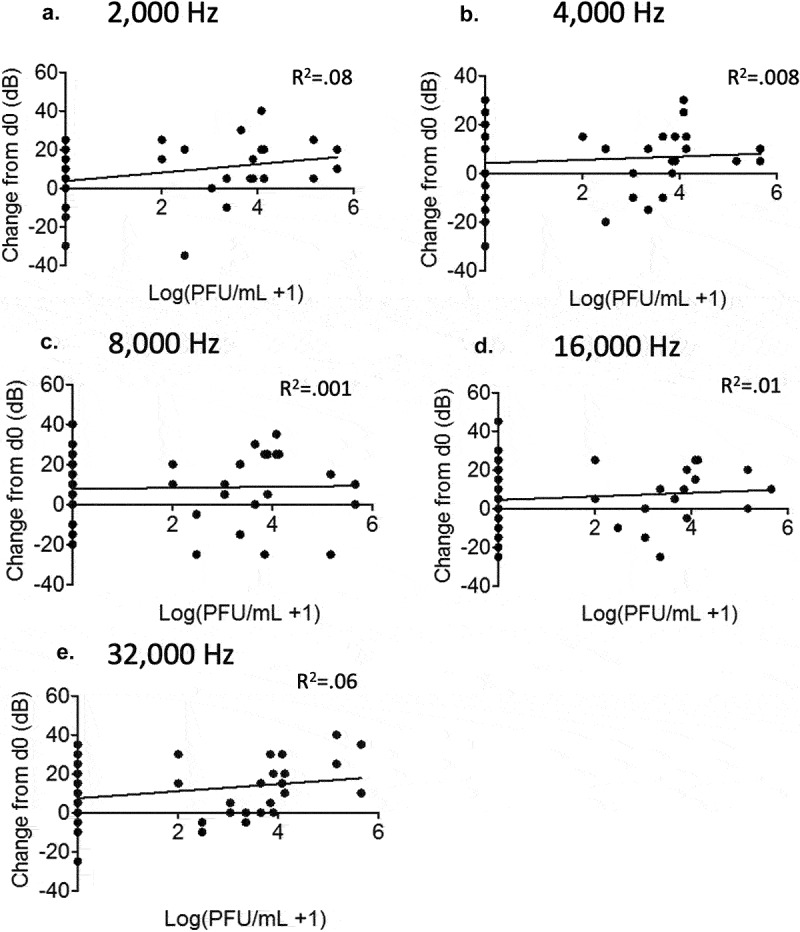
No correlation between viremia and hearing threshold levels in rP18-infected animals. Figures are reported as the changes in ABR thresholds from baseline decibel levels at day 0 for each rP18 cochlea tested at 2,000 Hz (a), 4,000 Hz (b), 8,000 Hz (c), 16,000 Hz (d), and 32,000 Hz (e) of sound and were compared to the serum’s viremia levels that were assessed at a time point when each ABR recording was taken. Statistical analyses were performed using linear regression, and the R^2^ values indicating no significant correlations were shown for each figure.

### Hearing loss occurred in a significant portion of the rP18-infected animals

To assess whether guinea pigs experienced hearing loss during rP18 infection, we recorded hearing levels by ABR for five sound frequencies at various timepoints following the infection. We found that, when averaged as a group, the rP18-infected animals had significantly increased hearing threshold levels post infection (when compared to their baseline levels) than the mock or rP2-infected groups (Figure 3). These trends were consistent across sound frequencies tested and were present in either or both ears of some of the animals, indicating either unilateral or bilateral hearing loss. Hearing loss could persist up to the terminal point in some of the rP18-infected animals (Table 1). Five out of eight (5/8) of the rP18-infected animals experienced hearing loss, while one out of eight (1/8) had less definitive evidence of hearing loss due to increased hearing thresholds occurring on nonconsecutive days (Table 1). However, it does not appear that hearing threshold levels significantly correlate with serum’s viremia levels in the rP18-infected animals (Figure 4). It is also noteworthy that whereas most of the rP2-infected animals cleared the infection, one of the rP2-infected animals showed a relatively low level of the virus present at day 8 of the infection, but this was likely insignificant in terms of disease pathogenesis as this animal fared well throughout the course of the infection, despite its inability to clear the infection at 8 days post infection when the serum sample was collected for the analysis. Altogether, our results indicate that a significant number of the rP18-infected animals experienced hearing loss during the acute course of the virus infection.

**Table 1.** Summary of hearing ability in individual rP2- or rP18-infected outbred Hartley guinea pig.

A. rP18			
Animal #	Hearing Loss- Left Ear (Day)	Hearing Loss- Right Ear (Day)	Peak Viremia (log(PFU/mL) and (Day)
1	-	+ (Day 4- Day 8)	ND
2	-	? (Day 8 and Day 16)	3.04 (Day 12)
3	-	-	2.48 (Day 8)
4	+ (Day 12)	-	5.16 (Day 12)
5	+ (Day 16)	+ (Day 12-Day 16)	4.13 (Day 16)
6	+ (Day 16)	+ (Day 12- Day 16)	ND
7	-	-	3.65. (Day 8)
8	+ (Day 4- Day 8)	+ (Day 4- Day 12)	5.65 (Day 12)
B. rP2			
Animal #	Hearing Loss- Left Ear (Day)	Hearing Loss- Right Ear (Day)	Peak Viremia (log(PFU/mL) and (Day)
1	-	-	ND
2	-	-	1.6987 (Day 8)
3	-	-	ND
4	-	-	ND
5	-	-	ND
6	-	-	ND

Charts summarize the days post infection in which hearing loss, which was determined as >/ = 20 dB increase in ABR threshold compared to baseline levels at day 0, was presented for each of the animals infected with either the rP18 (a) or rP2 (b) virus. One animal (rP18 animal #2) is marked with a question mark to indicate less definitive demonstration of hearing loss due to increased hearing thresholds occurring on nonconsecutive days. The dates (in parenthesis) in which the highest viremia levels were observed are also indicated, when testing for infectious virus levels in serum at 4-day intervals from day 4 to day 16 post infection for each animal. Viremia levels are reported as log(PFU/mL) or as non-detectable (ND).

### Animals with hearing loss did not demonstrate overt histopathological changes in the cochlea

**Figure 5.**
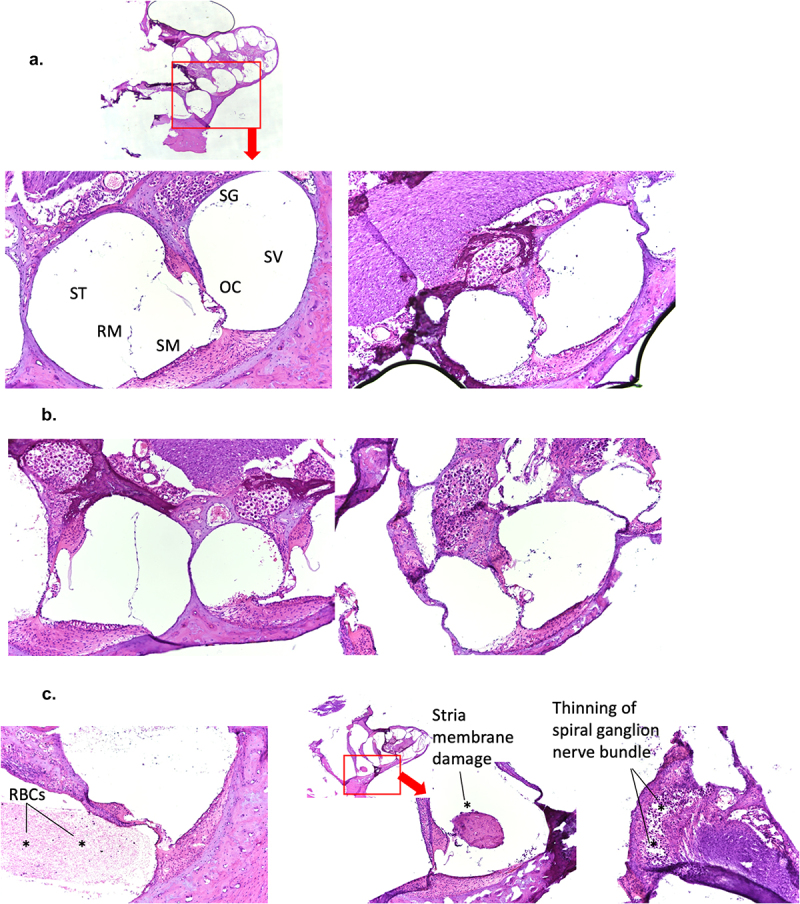
Lack of consistent cochlear pathology associated with hearing loss in virulent rP18-infected outbred Hartley guinea pigs.

Virus-induced hearing loss can have a number of causes ranging from temporary inflammatory ear-canal blockages to damages to the auditory nerves, hair cells, and/or support structures found in the cochlea [39,40]. To determine whether there were any definitive signs of inflammation or damages within the cochlea of the virulent rP18-infected animals, we processed cochleae from some of the rP2- and rP18-infected animals for H&E staining. In general, we did not find any obvious evidence of immune cell infiltration, inflammation, or overt damages to the auditory nerves or to other structures of the hearing-impaired rP18-infected cochleae (Figure 5(b)) when comparing to cochleae of rP18-infected animals that showed no evidence of hearing loss (Figure 5(a)). However, 3 of 11 tested cochleae of the rP18-infected animals with hearing loss showed evidence of internal hemorrhage, separation of a portion of the stria membrane and possible damage to the spiral ganglion nerves (Figure 5(c)), but these findings were not consistently observed in all hearing-impaired animals.

Hematoxylin and eosin (H&E) staining is shown for cryo-sectioned cochleae from non-hearing impaired (a) and hearing impaired (b) animals. Low-magnification images in (a and c) show the overall structures of guinea pig’s cochleae and the red boxes show the location of the higher magnified images. The scalar vestibuli (SV), scalar tympani (ST), stria membrane (SM), Reissner’s membrane (RM), the organ of corti (OC), and spiral ganglion (sg) nerve cell bundles are labeled in (a). (c) Occasional instances of potential cochlear abnormalities in some cochleae of animals infected with the virulent rP18 virus (asterisks), including the presence of red blood cells (RBCs) in the scala tympani (left), separation of a portion of the stria membrane (center) and possible thinning of the spiral ganglion nerve cell bundle (right) are also shown.

**Figure 6.**
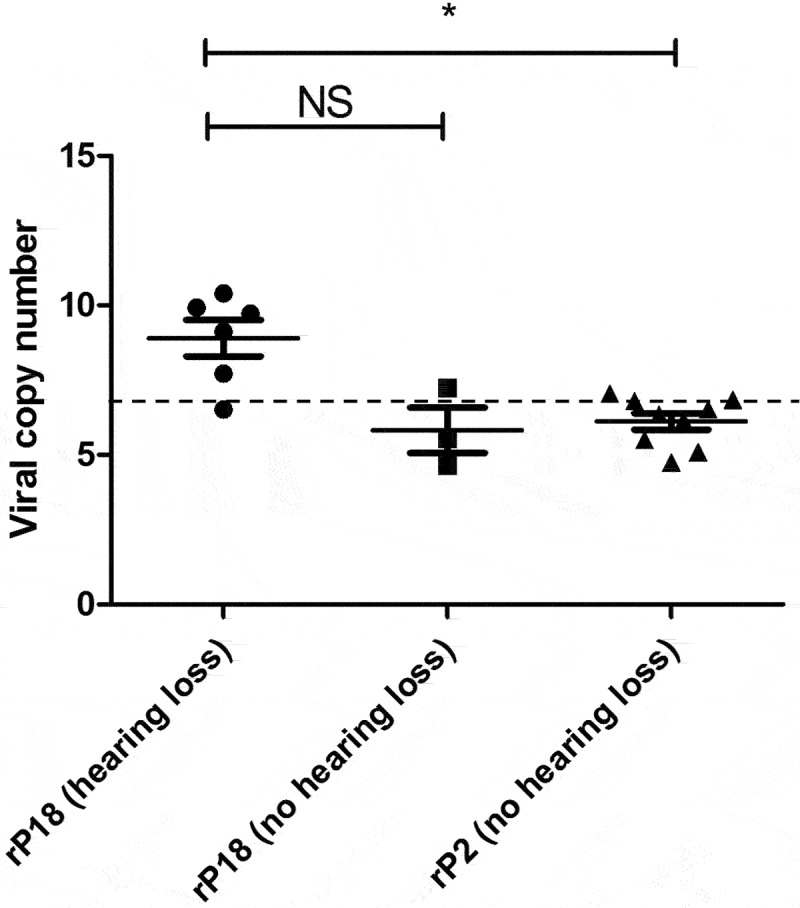
Presence of PICV RNAs in the cochlea of some rP18-infected animals with hearing loss. Frozen cochleae of rP2- and rP18-infected animals were homogenized and used to extract PICV RNAs for amplification by RT-qPCR. The absolute viral RNA copy numbers in the cochlea’s samples were determined based on a standard curve of DNA amplification of the PICV NP gene-containing plasmid. Data is reported for one of three RT-qPCR replicates, with similar results being found for the other replicates not shown. Statistical analyses were performed using Kruskal–Wallis one-way ANOVA. *=p < 0.05, **=p < 0.01, ***=p < 0.001, “NS” non-statistically significance.

### Presence of viral RNA in cochleae of some animals with hearing loss

As previous studies have determined that LASV can be detected in the cochleae of some of the LASV-infected animals [25,27] we wish to determine whether PICV RNAs could be present in some of the cochleae of outbred Hartley guinea pigs infected with either the rP2 or rP18 viruses. Toward this end, we extracted RNAs from some of the cochleae of those virus-infected animals for use to detect and quantify the levels of PICV genomic materials by quantitative RT-qPCR. The copy numbers of PICV RNA were found to be significantly higher in the cochleae of the rP18-infected animals with hearing loss than in the cochleae of the rP18-infected animals without hearing loss or of the rP2-infected animals (Figure 6). However, it is noteworthy that the viral copy numbers detected in the cochleae were relatively low, which makes it difficult to assess whether viral RNA presence in the cochlea plays a direct or indirect role in the development of hearing impairment in those animals. Similarly, a previous report showed that LASV antigen could only be detected in a portion of the fatally infected guinea pigs, which ranged from 1/6 to 3/6 animals depending on the region within the cochlea being analyzed [27].

## Discussion

There are some similarities and differences between the various animals used to model human LF in terms of hearing loss. A major challenge in working with LASV is that it is a BSL4-level pathogen that requires the necessary high biocontainment laboratory. As such, there have only been three studies done to date, to the best of our knowledge, to examine the important issue of hearing loss in LASV-infected animals [25–27]. Here, we describe the relevant findings reported in those studies as they pertain to those found in our current study.

Huynh and colleagues examined hearing loss in inbred strain 13/N guinea pigs [27] that are highly susceptible to lethal LASV infection and can develop a systemic infection characterized by liver damage, arthritis and interstitial pneumonia [24]. They infected either naïve 13/N guinea pigs or 13/N guinea pigs that had been vaccinated with LASV single-cycle replicon particles [41] subcutaneously with 1 × 10^4^ pfu of recombinant LASV-Josiah, and monitored them for up to 42 days post infection [27]. In our current study, the authors of this study did not find any consistent evidence of damages to the cochlea, but they did find the presence of LASV antigen in several cochlear structures (i.e. in 6/14 moribund animals), including the stria vascularis, Reissner’s membrane, and the capillaries and membranes supporting the organ of Corti [27]. LASV antigen was also found in blood vessels within or adjacent to the cochlea of 9/14 moribund animals [27]. They also noted the presence of swelling and degeneration of the temporalis muscle somewhat proximal to the ear canal. Interestingly, all animals that were vaccinated with LASV single-cycle replicon particles [41] either before or shortly after LASV infection did not succumb to the infection but showed perivascular and mononuclear inflammation of the soft tissues and muscles surrounding the cochlea in lieu of any LASV antigens being present there [27]. Unfortunately, no hearing loss was conducted in this study [27].

The findings of the presence of viral materials in the cochlea of some of the animals in our current study and in the published report [27] likely underscore the systemic nature of viral infection of the animals. It has previously been hypothesized that LASV infection most likely spreads to the inner ear through the blood vessels that innervate and supply the cochlea with blood and oxygen [25,42,43]. It is noteworthy that Cashman and colleagues noted significantly increased hearing threshold levels and the presence of perivascular inflammation in the tissues adjacent to the cochlea surrounding the auditory nerve in two of the three macaques infected with 1 × 10^3^pfu of LASV-Josiah intramuscularly (IM) as determined by the brainstem auditory evoked response communication (BAERCOM) method, which is similar to the ABR method used in our current study [26]. The authors of this study also noted inflammation of the vessels supplying blood to the cochlea and hypothesized that this could potentially starve the cochlea of oxygen and could therefore play a role in the development of hearing loss [44–51] despite LASV not being detected in the cochlea of these animals by immunohistochemistry (IHC) [26]. Unfortunately, no baseline BAERCOM readings were taken before LASV infection of these animals to ascertain whether it was indeed a causative effect of the hearing loss [26]. It is important to note that, similar to our finding, the authors of this study [26] did not find any other overt histopathological changes in the cochlea of these animals that can directly implicate them as an etiology of hearing loss.

In a separate study, Yun and colleagues used the STAT1 knock-out (KO) immunodeficient mice as a model for LASV infection, which differs from other immunodeficient mouse strains [24] in their levels of susceptibility to lethal infection with LASV as well as in the development of hepatic and splenic dysfunction, as is often seen in human LF patients [52]. STAT1 KO mice were infected systemically (IP) with 1 × 10^4^pfu or 1 × 10^5^pfu of LASV strains isolated from fatal (LV2384) and non-fatal (LV2350) human LF cases obtained during an outbreak in Sierra Leone in 2012 [25]. These virus-infected animals were monitored for 60 days post infection [25]. Hearing threshold levels were determined in mice before the infection and during the convalescence stage by observing behavioral changes, such as ear or body twitch in response to click stimuli [25]. All surviving mice infected with either of the two dosages of LV2384 or with 1 × 10^5^pfu LV2350 were designated as deaf, which was defined as showing no behavioral changes to two or more click stimuli, by day 60 postinfection. In comparison, none of the IFN α/βγ receptor KO mice infected systemically (IP) with 1 × 10^5^ pfu of LV2350 developed any signs of deafness by day 60 postinfection [25]. The authors quantified hearing loss by measuring the magnitude of movement in response to sound and found that hearing loss in the STAT1 KO mice appeared to be progressive starting from day 29 post infection [25]. Finally, they found a significant level of thinning of the vestibulocochlear nerve and of the stria vascularis, which is a vascularized epithelial layer surrounding the scalar media duct in the cochlea that produces endolymph fluid to fill the scala media, in the hearing-impaired STAT1 KO mice. They also found LASV antigen and CD3+ T cell staining overlapping with the areas of damage in the cochlea of some of the hearing-impaired animals [25]. The cochlea’s immune cell infiltrate in this study might explain the role of the observed nonspecific T cell infiltration into these tissues in some severe cases of human LF [53,54]. While this study has benefited from an increased sampling size by utilizing mice (instead of nonhuman primates) in ABSL4 laboratory and by finding clear evidence of cochlear pathology, it is noteworthy that immunodeficient animals were used in this study that might not authentically reflect the human conditions.

The findings of inflammation and vasculitis in or surrounding the cochlea either at the late stage of virus infection [25] or well after recovery from LASV infection [25,26] (e.g. from 29 days to 42 days post infection) suggest that hearing dysfunction in animals recovering from long-term LASV infection might be associated with chronic vascular dysfunction and blood flow interruption to the cochlea. These conditions, however, might not be reflected in our outbred Hartley guinea pigs that had been acutely infected with the virulent rP18 strain of PICV for only up to the terminal point of 16 days, thereby limiting observation of animals in the convalescent stage of infection. Additionally, the variable association between cochlea’s inflammation and hearing loss seen in these mammarenavirus-induced SNHL models [25–27] appears to be different than SNHL models caused by other viruses, such as human cytomegalovirus (CMV) [10,55–57] and rubella virus infections [58,59] where hearing loss is associated with a consistent level of immune cell infiltration [60]. Additionally, hearing loss caused by cytomegalovirus or rubella virus infection has consistently been associated with cochlear pathology including damage to the stria vascularis and the Reissner’s membrane, loss of hair cells, and fibrosis in the scala tympani [10,55–59]. It should be noted that hearing loss resulting from CMV and rubella viral infections is often associated with congenital infections, whose presentation may differ from LASV-associated hearing loss due to the ongoing maturation of the fetal immune system and/or of the inner ear during pregnancy [61].

In summary, in our current study, we found that a significant portion of outbred Hartley guinea pigs infected with the virulent rP18 PICV show acute hearing loss (Figures 2 and 3 and Table 1). However, hearing loss did not appear to be strongly associated with serum’s viremia levels (Figure 4) or cochlear pathology (Figure 5). On the other hand, viral RNAs were consistently detected in the cochlea of the virulent rP18-infected animals that experienced hearing loss (Figure 6). This is the first report on an immunocompetent small animal model of mammarenavirus-induced hearing loss that may be used to evaluate potential therapeutics against virus-induced hearing impairment under a conventional laboratory setting.

## Data Availability

The data set linked with this submission can be found at 10.6084/m9.figshare.19352432
